# Sleep fragmentation in critically ill children: a review of contributing factors in the pediatric intensive care unit and neurodevelopmental outcomes

**DOI:** 10.3389/frsle.2025.1629408

**Published:** 2025-08-26

**Authors:** Alana GaHyun Byeon, Shelly K. Weiss, Elaine Gilfoyle, Nicole K. McKinnon

**Affiliations:** ^1^Department of Medical Physiology, University of Toronto, Toronto, ON, Canada; ^2^Division of Neurology, Department of Paediatrics, The Hospital for Sick Children, Toronto, ON, Canada; ^3^Department of Paediatrics, Temerty School of Medicine, University of Toronto, Toronto, ON, Canada; ^4^Department of Critical Care, The Hospital for Sick Children, Toronto, ON, Canada; ^5^Program in Neurosciences and Mental Health, Hospital for Sick Children Research Institute, Toronto, ON, Canada

**Keywords:** sleep fragmentation, delirium, neurodevelopmental outcomes, Post-Intensive Care Syndrome (PICS), intensive care units, pediatric

## Abstract

Sleep is a critical neurobiological process essential for brain maturation, emotional regulation, cognitive development, and overall organ system homeostasis. In the pediatric intensive care unit (PICU), sleep architecture is frequently disrupted by environmental stimuli, sedation, and clinical interventions, resulting in sleep fragmentation. Unlike sleep deprivation, sleep fragmentation preserves sleep duration but impairs its continuity and depth, disproportionately affecting slow-wave sleep, that is essential for growth, healing, in addition to immune function and REM sleep, that is fundamental for synaptic plasticity, neurogenesis, and memory consolidation. These disruptions are particularly concerning in children, who require more sleep than adults due to ongoing neurogenesis and rapid somatic growth, rendering them uniquely vulnerable to adverse effects. Emerging evidence links fragmented sleep in the PICU to altered neurodevelopmental trajectories and increased risk of Pediatric Post-Intensive Care Syndrome (PICS-p), with delirium serving as a key mediator. Despite promising adult studies on sleep-promoting interventions and EEG-based monitoring, pediatric research remains limited. Future research should prioritize objective sleep monitoring, developmental tailoring of care protocols, and longitudinal studies to clarify the impact of sleep fragmentation on recovery and neurodevelopment. This narrative review highlights the urgent need to recognize and preserve sleep as a modifiable determinant of neurocognitive outcomes in critically ill children.

## 1 Introduction

Sleep is a dynamic and fundamental physiological process essential for neurodevelopment, cognitive maturation, and overall health. Sleep evolves over a lifetime in support of age-specific biological needs. The ultradian cycle of sleep describes 24-h sleep-wake cycles. Within the period of sleep, cycles of stages of non-rapid eye movement (NREM) and rapid eye movement (REM) occur, with the time spent in each dependent on age. Slow-wave sleep (SWS), the deepest phase of NREM sleep, is critical during early life for growth hormone secretion, cellular repair, and immune regulation, while REM sleep facilitates emotional regulation, memory consolidation, and neural plasticity (Soto et al., 2024). Together, these stages form the architecture of restorative sleep, which makes up a substantial portion of sleep in childhood in support of growth, immune system maturation, and brain development.

The integrity of this architecture is frequently disrupted in children cared for in intensive care units (ICUs), where continuous monitoring and interventions generate environmental and physiological stressors that profoundly alter circadian rhythms and sleep continuity (Boyko et al., 2017). Unlike total sleep deprivation, sleep fragmentation—marked by recurrent arousals and incomplete sleep cycles—can result in non-restorative sleep even when total sleep duration appears preserved (Hassinger et al., 2023). In adult ICU patients, such disruption has been linked to adverse outcomes including delirium, cognitive dysfunction, prolonged hospitalization, and increased morbidity (Boyko et al., 2017; Herrup et al., 2017; Knauert et al., 2018).

Despite these well-documented associations in adults, sleep fragmentation remains an underexplored determinant of recovery in pediatric critical care. Children admitted to the pediatric intensive care unit (PICU) are uniquely susceptible to the consequences of sleep fragmentation due to their developmental dependence on high proportions of SWS and REM sleep (Berger et al., 2021). Admission to the PICU often results in significant alterations in sleep patterns, driven by a combination of PICU-related stressors such as environmental noise, lighting, sedation, and mechanical ventilation (Kudchadkar et al., 2019; Greenfield et al., 2020; Stremler et al., 2021). On top of this, children's reliance on caregivers, limited communication capacity, and developmental heterogeneity complicate both assessment and management of sleep disruption (Burger et al., 2024). Consequently, while these children are uniquely vulnerable, awareness of modifiable contributors to sleep fragmentation remains limited.

Given the significant role of sleep in neurodevelopment and recovery, identifying and mitigating these disruptions is crucial. This narrative review synthesizes emerging evidence on sleep fragmentation in critically ill children, examines its links to neurocognitive and emotional impairments, and highlights strategies to reduce fragmentation and protect sleep as a modifiable factor in neuroprotective critical care.

For this review, we conducted an iterative search, starting broadly, examining the relationship between sleep and neurocognitive outcomes in children. We then focused our search on critically ill patients, including adults, as there is very little in the pediatric literature. The senior authors run their unit's PICU outcomes clinic and have significant knowledge related to PICS-p. Utilizing this background, we finally attempted to delve into the PICS-p literature and determine if sleep was being investigated as a modifiable risk factor for improving outcomes after pediatric critical illness.

## 2 Sleep and its neurodevelopmental significance

Sleep and wake follow a 24-h ultradian cycle, regulated by two key mechanisms that control wakefulness and sleepiness. The homeostatic process increases the propensity for sleep as wakefulness is prolonged, while the circadian system—driven by the suprachiasmatic nucleus—modulates sleep propensity across the 24-h cycle, promoting alertness during the day and facilitating sleep onset in the evening. Together, these mechanisms orchestrate the timing and depth of sleep to maintain physiological and cognitive equilibrium (Desai et al., 2024; Shakankiry, 2011).

Sleep itself is a complex, dynamic process composed of cyclic transitions between non-rapid eye movement (NREM) and rapid eye movement (REM) sleep. NREM is subdivided into three stages (N1–N3), with N3—also known as slow-wave sleep (SWS)—representing the deepest and most restorative phase (Memar and Faradji, 2018). Each stage is characterized by specific electroencephalographic (EEG) features that reflect underlying neuronal oscillations and cortical synchronization. NREM sleep, particularly SWS, is implicated in growth hormone secretion, energy restoration, and immune system development (Wilckens et al., 2018). REM sleep plays a crucial role in neuronal growth, sensorimotor integration, memory consolidation and emotional regulation (Zhou et al., 2020). Collectively, these stages synergize to facilitate neurodevelopmental processes, particularly in early life when the brain undergoes rapid structural and functional maturation (Ribeiro et al., 2004) (Table 1). The emergence and refinement of sleep architecture follow a developmental trajectory shaped by neurobiological maturation, hormonal regulation, and behavioral routines. The development of stable circadian rhythms and sleep regulation begins *in utero*, with sleep patterns adapting across childhood and adolescence to modulate the timing, depth, and duration of sleep (Escobar et al., 2021; Bathory and Tomopoulos, 2017).

**Table 1:** An overview of sleep stage distribution across pediatric developmental stages, highlighting expected sleep composition, neurophysiological characteristics, and their implications for cognitive, emotional, and somatic development.

**Type of sleep**	**Age group**	**Amount expected**	**Physiological characteristics**	**Neurodevelopmental implications**	**Consequences of disruption**	**References**
Light sleep (NREM stage 1 & 2)	Neonate (0–1 month)	20–50%	Sleep spindles: absent; normally characterized by 12–14 Hz oscillatory bursts during sleep. Trace alternant pattern: most frequent neonatal EEG pattern; marked by intermittent high-amplitude slow waves mixed with faster activity. Typically disappears by 2 weeks of age and absent by 6 weeks.	Lays foundation for circadian rhythm development and future sleep consolidation.	Linked to heightened stress response, somatic pain, impaired cognition, emotional distress, mood disorders, and reduced quality of life. Disturbs maturation of sleep-wake cycles and neural network organization.	Goel and Goel, 2024; Lenehan et al., 2023; Grigg-Damberger et al., 2007; Barclay and Gregory, 2013; Mirmiran et al., 2003
	Infant (1–12 months)	25–50%	Sleep spindles and K-complexes emerge by 3 months, enhancing sleep stability.	Facilitates early memory formation, sensorimotor integration, and establishment of sleep homeostasis.	Disruption may lead to heightened sensory reactivity, arousal disorders, and delayed motor milestones.	Goel and Goel, 2024; Lenehan et al., 2023; Grigg-Damberger et al., 2007; Jiang, 2020
	Early childhood (1–5 years)	50–60%	Increased N1/N2 time due to reduced REM/SWS proportion; strong sleep spindle presence.	Sleep spindles contribute to motor learning and language development.	Disruption may impair procedural learning, increase sleep fragmentation, and contribute to attentional lability.	Goel and Goel, 2024; Barclay and Gregory, 2013; Carskadon and Dement, 2011; Fogel and Smith, 2011
	Late childhood (6–12 years)	50–55%	N2 becomes the predominant stage of sleep; increased spindle density.	Helps integrate daily experiences and primes brain for higher-order tasks.	Associated with attention deficits, learning difficulties, school struggles, and risk-taking behaviors.	Goel and Goel, 2024; Barclay and Gregory, 2013; Carskadon and Dement, 2011; Paruthi et al., 2016; Fogel and Smith, 2011
	Adolescent (13–18 years)	55–60%	Light sleep dominates due to SWS decline; brain remains more reactive to external stimuli.	Maintains continuity of sleep cycles, supports emotional regulation and transition to adult-like sleep architecture.	May affect academic performance, daytime alertness, and sleep efficiency.	Goel and Goel, 2024; Barclay and Gregory, 2013; Anastasiades et al., 2022; Tarokh et al., 2016; Fogel and Smith, 2011
Deep sleep (NREM stage 3—SWS)	Neonate (0–1 month)	Absent	Predominantly active (REM) and quiet sleep; absence of slow-wave activity.	Early sleep stages support initial neural circuit maturation critical for later development; brainstem-driven regulation dominates.	N/A	Goel and Goel, 2024; Grigg-Damberger et al., 2007
	Infant (1–12 months)	Gradual emergence	Slow-wave emergence: development of NREM stages 3 and 4 around 3–5 months; characterized by delta brain activity (0.5–4 Hz).	Facilitates early synaptogenesis, early memory formation, and sensorimotor integration	Linked to impaired attachment formation, hyperactivity, motor development, and altered stress response.	Goel and Goel, 2024; Lenehan et al., 2023; Grigg-Damberger et al., 2007; Jiang, 2020
	Early childhood (1–5 years)	Increase	High increase in SWS EEG shows high-amplitude, low-frequency delta waves	Critical for language acquisition, executive function development, synaptic pruning begins	May impair emotional regulation and early learning; associated with increased risk of behavioral dysregulation	Goel and Goel, 2024; Barclay and Gregory, 2013; Kurth et al., 2010; Cauter and Plat, 1996
	Late childhood (6–12 years)	Peak	Proportion of SWS is greatest; high delta power and cortical synchronization Supports rapid physical growth and neurocognitive development	Promotes memory consolidation, cortical network integration, and somatic growth (via growth hormone secretion). Strengthens neural pathways linked to learning and skill acquisition.	May cause cognitive impairment, behavioral challenges, weakened immune response, and growth delays.	Goel and Goel, 2024; Barclay and Gregory, 2013; Carskadon and Dement, 2011; Kurth et al., 2010; Cauter and Plat, 1996
	Adolescent (13–18 years)	Gradual decline	SWS declines ~40% during adolescence, reflecting synaptic refinement. Despite high physiologic demand (e.g., growth hormone), N3 duration shortens.	Maturation of emotional regulation and executive functions during adolescence. Strengthens prefrontal cortex circuits essential for decision-making and self-regulation.	Reduced SWS associated with mood disorders, impulsivity, and risk-taking behaviors.	Goel and Goel, 2024; Barclay and Gregory, 2013; Anastasiades et al., 2022; Tarokh et al., 2016; Kurth et al., 2010
REM Sleep	Neonate (0–1 month)	50–80%	50–60 min NREM-REM cycles; high REM proportion compared to adults.	High REM promotes synaptogenesis and formation of neural circuits, supporting information acquisition and memory consolidation in an environment with minimal external stimulation.	Risk of impaired sensory processing and abnormal neural connectivity.	Goel and Goel, 2024; Grigg-Damberger et al., 2007; Barclay and Gregory, 2013; Lockley and Foster, 2012
	Infant (1–12 months)	30–40%	Decrease in REM sleep percentage; NREM-REM cycle lengthens.	Supports visual system development, memory consolidation and learning; critical for language development.	Linked to impaired cognitive development and emotional dysregulation.	Goel and Goel, 2024; Lenehan et al., 2023; Grigg-Damberger et al., 2007; Jiang, 2020
	Early childhood (1–5 years)	25–30%	REM cycles lengthen, become more stable and organized.	Supports emotional processing and regulation of affective responses	May impair socioemotional development, learning readiness, and sleep-related anxiety	Goel and Goel, 2024; Barclay and Gregory, 2013; Jiang, 2020; Jenni and Dahl, 2008; Paruthi et al., 2016
	Late childhood (6–12 years)	20–25%	Stabilization of sleep architecture: mature REM-NREM cycles (~90 min)	Involved in consolidation of complex emotional memories and problem solving.	Associated with anxiety, learning inefficiencies, and maladaptive emotional reactivity	Goel and Goel, 2024; Barclay and Gregory, 2013; Carskadon and Dement, 2011; Tarokh et al., 2016; Jenni and Dahl, 2008
	Adolescent (13–18 years)	20–25%	REM architecture stabilizes; REM density increases toward adulthood	Supports integration of new social and emotional experiences; critical for identify development, stress coping, and complex emotion regulation.	Linked to mood disorders, cognitive impairments, and decreased academic performance.	Goel and Goel, 2024; Barclay and Gregory, 2013; Anastasiades et al., 2022; Tarokh et al., 2016; Jenni and Dahl, 2008

As infants mature, REM sleep decreases while SWS increases, peaking during late childhood and adolescence when growth hormone secretion and sexual maturation are most active. This stage supports both neurodevelopmental and physiologic processes such as immune regulation, tissue repair, and homeostatic balance.

**Figure 1:**
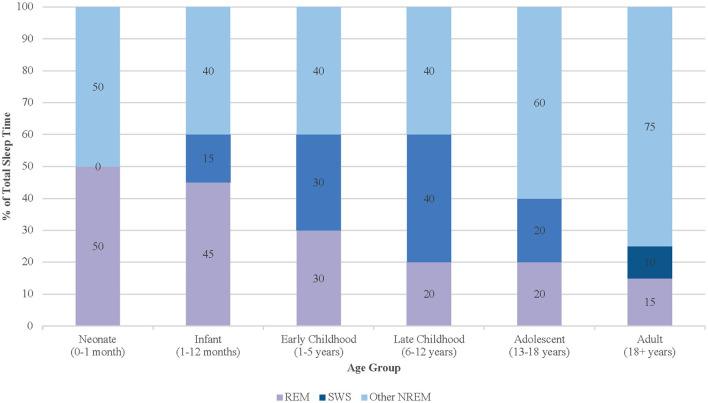
Age-related changes in sleep stage distribution from infancy through adulthood, with peaks in SWS during late childhood.

Compared to adults, children spend a markedly higher proportion of sleep in REM and SWS, highlighting their greater physiological requirement for restorative sleep (Figure 1). In neonates, REM sleep may constitute up to 50% of total sleep time, contributing to cortical differentiation and sensorimotor pathway development—particularly in the context of limited environmental stimulation (Dumoulin Bridi et al., 2015; Peirano and Algarín, 2007). By early childhood, the total time in REM sleep decreases while SWS increases, reflecting heightened growth velocity and memory consolidation demands. Current pediatric sleep research consistently shows that SWS and slow-wave activity (SWA) peak in late childhood, prior to puberty, and decline steeply during adolescence despite continued physiologic needs such as growth hormone secretion (Kurth et al., 2010). As adolescents complete puberty, SWS continues to decline toward adult levels, mirroring broader processes of cortical thinning and synaptic remodeling (Lockley and Foster, 2012; Feinberg and Campbell, 2010). These developmental dynamics in the different sleep states render pediatric populations uniquely sensitive to disruptions in sleep continuity and depth. Shortened ultradian cycles, higher REM density, and the predominance of SWS in early life collectively imply that even mild sleep fragmentation may have amplified neurodevelopmental consequences. Fragmented sleep has been associated with deficits in executive function, attentional regulation, and socioemotional processing in children, suggesting that maintaining sleep integrity is critical to optimizing developmental trajectories (Kurth et al., 2015).

The integrity of sleep architecture is essential for optimizing neurodevelopmental trajectories. Unlike total sleep deprivation, which primarily reduces sleep duration, sleep fragmentation disrupts the continuity and structure of sleep by causing frequent arousals that prevent the progression through normal sleep stages and result in incomplete cycles (Soto et al., 2024). Although total sleep deprivation leads to broad cognitive and physiological impairments, these effects can often be partially reversed with subsequent recovery sleep. In contrast, fragmented sleep allows for adequate sleep duration but disrupts its restorative functions, resulting in recurrent arousals, disrupted cycles, and reduced SWS and REM sleep (Bonnet and Arand, 2003).

As a result, this selective disruption of key sleep stages may render fragmentation even more detrimental than sleep deprivation. In adolescents, fragmented sleep and chronic sleep restriction have been linked with impairments in memory consolidation, executive function, and academic performance (Carskadon et al., 2004). Even mild disturbances in sleep continuity have been associated with deficits in cognitive processing, attention regulation, and socioemotional development (Yorbik et al., 2014). Moreover, disrupted sleep impairs emotional face recognition and inhibitory control, likely due to reductions in REM and slow-wave sleep and altered activity in inhibition- and vision-related brain regions (Lee et al., 2022). These findings collectively underscore that sleep fragmentation may not only compromise immediate cognitive functioning but may also interfere with long-term neurodevelopmental trajectories essential for behavioral and psychosocial outcomes.

## 3 Contributing factors

The PICU environment presents a range of intrinsic challenges to preserve sleep, with noise and lighting among the most prominent disruptors. Noise levels in the PICU regularly exceed World Health Organization guidelines of 35 decibels (dBA), often peaking above 90 dBA due to alarms, staff activity, and machinery (Romagnoli et al., 2020). Among various unwanted noise contributors, medical alarms were previously identified as the loudest, disruptive noises leading to discomfort, with medical equipment noise being the second highest (Kaur et al., 2016). Medical equipment alarms, while designed to enhance patient safety, contribute significantly to excessive noise levels within the ICU environment. The high frequency and unpredictability of these alarms can result in alarm fatigue, cognitive overload, and workflow interruptions among healthcare providers, potentially diminishing clinical efficiency and attentiveness to critical patient needs. Prolonged exposure to alarm noise may also exacerbate stress and disrupt both patient and staff wellbeing (Bridi et al., 2014).

In addition to auditory disturbances, artificial lighting in the PICU significantly disrupts circadian alignment, making it harder for children to fall and stay asleep. The circadian system regulates the timing of physiological processes across multiple organ systems, including the gastrointestinal tract, immune function, and metabolic regulation (Hassinger et al., 2023). In the PICU, median nocturnal light levels are typically low (around 4 lux), but care activities and monitor lights can cause peaks above 30–50 lux overnight, which may suppress melatonin secretion and disrupt circadian rhythms. Conversely, daytime light levels are often suboptimal for circadian entrainment, with median values around 27 lux—well below the 100–500 lux recommended for essential physiological function—highlighting the importance of both the timing and intensity of light exposure in pediatric critical care settings (Greenfield et al., 2020; Dervan et al., 2022). These findings have informed best practices in adult ICUs, but their applicability to pediatric patients is limited due to age-dependent differences in chronotype, dim light melatonin onset (DLMO), and photoreceptor maturation. For example, standardized ICU lighting routines (e.g., lights out at 9 PM) misalign with both early chronotypes in infants and delayed ones in adolescents, exacerbating circadian disruption regardless of total sleep time or fragmentation (Greenfield et al., 2020).

Therapeutic interventions, though necessary for stabilizing patients, also contribute substantially to disrupted sleep. Sedation and mechanical ventilation are significant contributors to disrupted sleep patterns, with profound implications for neurocognitive recovery and long-term outcomes (Al-Samsam and Cullen, 2005). Commonly used sedatives—including benzodiazepines, opioids, ketamine, barbiturates, and alpha-2 agonists —are frequently administered to manage pain, anxiety, and agitation in critically ill children. While these medications can prolong total sleep duration, they significantly alter sleep architecture, reducing restorative SWS and REM sleep (Kudchadkar et al., 2014). Benzodiazepines, for example, have been shown to decrease REM sleep while increasing lighter N2 sleep, leading to fragmented and non-restorative sleep (Zhao et al., 2022). Opioids, commonly used in combination with benzodiazepines, further suppress SWS and REM sleep, exacerbating sleep fragmentation (Al-Samsam and Cullen, 2005). Paradoxically, increasing sedative doses to improve perceived sleep quality often leads to negative impact on patient outcomes such as prolonged mechanical ventilation, agitation and further deterioration of sleep architecture (Shajan et al., 2023). Prolonged EEG studies have demonstrated that sedated children exhibit slower brain activity during sleep, characterized by diffuse delta wave activity and loss of N1 and REM stages. In a continuous EEG monitoring study of mechanically ventilated children, deeper levels of sedation were associated with greater disruptions in sleep architecture, including a higher proportion of REM stage loss and a more substantial decrease in N2 sleep compared to lighter sedation levels (Zhao et al., 2022).

Mechanical ventilation introduces additional challenges to sleep in the PICU. Patient-ventilator dyssynchrony disrupts sleep continuity and often necessitates higher sedative dosing, compounding sleep disturbances (Zhao et al., 2022). Polysomnography (PSG) studies have shown that mechanical ventilation reduces sleep efficiency and alters slow-wave activity, with younger children being particularly susceptible to these disruptions (Sanchez et al., 2022). Moreover, sleep spindles—hallmarks of memory consolidation during N2 sleep—are frequently diminished in this population, contributing to impaired cognitive recovery. Even after sedation is weaned, abnormalities in circadian rhythm and sleep continuity can persist, reflecting the long-term impact of ICU-related sleep disruption (Oxlund et al., 2023).

Routine caregiving activities represent another major contributor to sleep fragmentation. PICU patients may experience 40–60 care-related interruptions per night for tasks such as vital sign measurements, medication administration, suctioning, repositioning, and diagnostic procedures such as radiographs and phlebotomy (Zhao et al., 2022; Kalvas et al., 2023; Kalvas and Harrison, 2024; Witte et al., 2023). These interruptions often coincide with critical sleep stages, preventing children from completing full sleep cycles and reducing the proportion of restorative SWS and REM sleep. Nursing care procedures have been shown to increase pain and stress, further exacerbating sleep fragmentation. In adult ICU populations, frequent nursing interventions have been associated with increased N1 stage sleep and decreased N3 and REM sleep, patterns that are likely mirrored in pediatric patients (Zhao et al., 2022). Early morning and late evening rounds by physicians and trainees also contribute to sleep disruption, as these activities often occur during periods when children would otherwise be in deeper stages of sleep (Witte et al., 2023).

Parental insights further highlight key disruptors of pediatric sleep. Surveys consistently report pain, illness-related fear, and anxiety as the most cited causes of poor sleep in PICU patients (Kaur et al., 2016). Other significant factors include the inability to sleep at home, alarms from medical equipment, and disruptions to normal sleep schedules. Suctioning and breathing treatments, which are often necessary for children with respiratory conditions, were also identified as major disruptors. Interestingly, factors such as repositioning by nurses, ambient light, and hallway conversations were considered less disruptive by parents (Kaur et al., 2016; Hassinger et al., 2024; Nenningsland et al., 2024). These findings underscore the value of caregiver perspectives in identifying high-yield intervention targets for sleep optimization in the PICU, particularly in reducing procedural disruptions and improving environmental conditions.

## 4 Inflammation's role in sleep fragmentation and delirium

Sleep fragmentation in critically ill children triggers a cascade of pathophysiological and neurodevelopmental consequences (Bertrand et al., 2020). In the PICU, fragmentation is quantifiable using the Sleep Fragmentation Index (SFI), which measures the number of arousals or transitions to lighter sleep stages per hour of sleep (Gregory et al., 2021). Elevated SFI scores are common among critically ill children, driven by environmental stressors, autonomic instability, mechanical ventilation, and prolonged sedation (Alegria et al., 2023). Although total sleep duration may be preserved—or even increased in adolescents—this sleep is often highly fragmented and non-restorative, with marked reductions in both REM and SWS (Dervan et al., 2022). Sleep disruption also varies by age: infants and toddlers exhibit the greatest total sleep loss compared to home, while adolescents may accrue more sleep but with reduced quality. Caregiver-reported environmental disruptions, including alarm noise, suctioning, and inconsistent light-dark cycles, are associated with poorer sleep quality, as reflected in higher Survey of Sleep Quality in the Pediatric Intensive Care Unit (SSqPICU) scores (Hassinger et al., 2024).

Sleep fragmentation alters cytokine regulation, promoting a pro-inflammatory state that disrupts normal neurodevelopmental trajectories (Berger et al., 2021). Neuroinflammation has been implicated in structural and functional changes in the hippocampus and prefrontal cortex—regions critical for learning, memory, executive function, and emotional regulation (Bertrand et al., 2020). Repeated disruptions to SWS may impair synaptic downscaling necessary for neural reorganization and memory consolidation, contributing to persistent deficits in attention, memory, and affect regulation (Kurth et al., 2015; Zamore and Veasey, 2022). Similarly, reduced REM sleep compromises synaptic plasticity and has been linked to affective disorders such as anxiety, depression, and post-traumatic stress (Herrup et al., 2017). These effects are especially concerning during early childhood, when neuroplasticity is heightened and brain networks are rapidly reorganizing (Peirano and Algarín, 2007).

The long-term impact of sleep fragmentation is further supported by a prospective cohort study by Deng et al., which found that higher SFI scores in infancy and toddlerhood were associated with increased emotional and behavioral difficulties at age five. Children with higher SFI scores exhibited significantly elevated hyperactivity and total difficulty scores, even after adjusting for confounders (Deng et al., 2023). These findings underscore the enduring influence of early sleep fragmentation on emotional regulation and behavior, reinforcing its potential role in the development of PICS-p-related impairments. While the biological mechanisms linking sleep fragmentation to neurodevelopment are well-characterized, direct empirical evidence in PICU survivors remains limited.

Delirium is a frequent complication in pediatric critical illness and further compounds the neurodevelopmental consequences of sleep fragmentation. Sleep disruption is a major precipitant of delirium, as disrupted circadian signaling, increased nighttime arousals, and fragmented REM sleep contribute to altered consciousness and cognitive confusion (Gregory et al., 2021; Calandriello et al., 2018; Weatherhead et al., 2021). Children who develop delirium in the PICU are more likely to experience severe and persistent deficits across multiple PICS-p domains, including cognitive dysfunction, emotional dysregulation, and impairments in adaptive behavior (Kamat and Berkenbosch, 2021; Ramnarain et al., 2023).

Circadian disruption is a hallmark of pediatric delirium, suggesting that ICU-related sleep fragmentation is central to its onset and severity (Kalvas et al., 2023). Importantly, sleep fragmentation and delirium appear to have a bidirectional relationship: sleep loss predisposes children to delirium, while agitation and hyperarousal associated with delirium further disturb sleep architecture (Pisani and D'Ambrosio, 2020). A recent cohort study found that nearly 25% of PICU patients developed delirium, with risk factors including neurological comorbidities and liver dysfunction. Delirium was independently associated with increased PICU length of stay, higher mortality risk, and poorer sleep quality. Notably, sedative regimens influenced delirium risk, with benzodiazepines linked to higher incidence and the role of dexmedetomidine remaining equivocal (Lei et al., 2023).

The pathophysiology of delirium remains poorly understood, but likely involves complex interactions between premorbid conditioning, underlying disease state(s), and the intensive care environment (Fan et al., 2024). Similar to sleep fragmentation, increased inflammatory markers (Il-6, Il-8, IL-10, IL-18, and TNFa) have been reported in patients with delirium. Additionally, elevations in biomarkers of microglial activation (S-100B) and neuronal injury (neuronal-specific enolase) positively correlated with delirium severity (Fan et al., 2024; Anderson et al., 2016; van den Boogaard et al., 2011; Plaschke et al., 2010; Lammers-Lietz et al., 2022; McNeil et al., 2019; Hughes et al., 2018; Brummel et al., 2024). This has led to a proposed mechanism involving the crossing of inflammatory mediators into the brain through a leaky blood-brain barrier, resulting in the functional impairments observed with delirium (Smith et al., 2024; Heffernan et al., 2025). This, however, does not fully account for the significant role the PICU environment has been shown to play in PICU delirium.

Beyond the ICU stay, delirium has been associated with sustained declines in health-related quality of life (HRQL). Dervan et al. reported that delirium independently predicted HRQL deterioration, particularly in psychosocial functioning (Dervan et al., 2022). Similarly, Silver et al. demonstrated that pediatric delirium corresponded with lower parental perception of their child's quality of life (QOL) up to one-month post-discharge. While causality remains unclear, baseline HRQL scores were comparable between groups, strengthening the argument that delirium itself contributes to long-term QOL impairments (Silver et al., 2020). Additionally, the psychological toll of delirium on families is substantial; parents frequently report emotional distress, increased anxiety, and altered perceptions of their child's recovery (Thibault et al., 2024). These findings highlight the need to address delirium not only as a neuropsychiatric complication, but as a multifactorial condition with far-reaching implications for child and family outcomes.

## 5 Pediatric Post-Intensive Care Syndrome (PICS-p)

Over the past several decades, advances in critical care have enabled the field to broaden its focus beyond survival to minimize morbidity and improve long-term outcomes. While Post-Intensive Care Syndrome (PICS) has been well-established in the adult population (Bouzgarrou et al., 2024; Hiser et al., 2023; Ahn et al., 2025; Zhou et al., 2023; Inoue et al., 2024; Schwitzer et al., 2023), pediatric-specific PICS has only recently been conceptualized. First introduced by Manning et al. (2018), PICS-p has gained recognition as a critical area of research in pediatric critical care, encompassing aspects of the post-ICU experience that are unique to children and their families.

PICS-p is a multifaceted condition characterized by newly acquired or worsened impairments in physical, cognitive, emotional, and social functioning following discharge from the PICU (Manning et al., 2018). As survival rates for critically ill children have improved, attention has shifted to the long-term outcomes of PICU survivors, revealing significant morbidity and impaired quality of life despite advances in critical care (Long and Fink, 2021; Quadir et al., 2024). PICS-p encompasses a spectrum of challenges that extend beyond the acute phase of illness, affecting children and their families for months to years after hospitalization.

Cognitive impairments are among the most debilitating consequences of PICS-p, affecting intelligence, memory, attention, and executive function (Kaur et al., 2016). Children with a history of PICU admission demonstrated significantly poorer performance across multiple neurocognitive domains—including working memory, processing speed, executive function, and sustained attention—as well as lower daily living skills and increased internalizing problems, compared to both normative and non-PICU samples (Chaiyakulsil et al., 2020; Canavera et al., 2023; Als et al., 2013). This highlights the disproportionate burden of neurodevelopmental impairment following critical illness, independent of PICU disease severity. A major challenge in understanding cognitive outcomes is the presence of pre-existing neurodevelopmental delays in many children requiring intensive care. Direct assessments have revealed new neurological and neurocognitive concerns in 30% of PICU survivors, with caregiver reports corroborating widespread concerns in cognition and emotional wellbeing (Hall et al., 2022).

Emotional and behavioral challenges are prevalent among PICU survivors, with up to 25% of children experiencing psychological difficulties within the first year after discharge (Rennick et al., 2014). The prevalence of post-traumatic stress disorder (PTSD) and major depressive disorder in PICU survivors is particularly concerning, with estimates as high as 28% and 13%, respectively (Davydow et al., 2010). Following PICU discharge, children commonly experience increased anxiety and reduced self-esteem as well as persistent sleep disturbances within 2 months post-discharge. Additionally, nearly half of children exhibited delays in personal-social functioning, indicating broader psychosocial impairments that may contribute to long-term emotional dysregulation and behavioral difficulties (Ducharme-Crevier et al., 2021).

Most importantly, the impact of PICS-p extends beyond the child, affecting families' psychological wellbeing, financial stability, and overall quality of life. Parents of PICU survivors often experience fear, uncertainty, and emotional distress during the early phases of their child's illness, with many feeling unprepared for the challenges of post-discharge care (Kirk et al., 2015). Increased family burden scores after PICU discharge were associated with worsening child functional status in several studies, creating a bidirectional relationship between the child's impairments and family stress (Meert et al., 2016). Financial burdens, including medical expenses and lost income, further compound the challenges faced by families. Despite these difficulties, some families report post-traumatic growth, finding meaning and resilience in the face of adversity (Rodríguez-Rey and Alonso-Tapia, 2017). Importantly, the subdomain of family functioning intersects meaningfully with all other PICS-p domains, highlighting its centrality in shaping recovery trajectories.

Researchers are actively trying to understand factors that increase children's risk for PICS-p, in addition to evaluating interventions to mitigate this risk. Currently, several longitudinal cohort studies are ongoing, including a large, multi-site study in the United States involving 30 PICUs (Curley et al., 2024b). The current recommended core outcome measurements to standardize the assessments used in PICS-p research (Pinto et al., 2022) do not include in-depth assessments of sleep hygiene beyond evaluation of nightmares amongst some of the pediatric trauma screens (Sachser et al., 2017). Given the importance of sleep to neurocognitive and emotional development, we suggest that additional screening for disordered sleep, utilizing a validated measure such as the Sleep Disturbance Scale for Children (SDSC) (Bruni et al., 1996) or the Children's Sleep Habits Questionnaire (Dias et al., 2018), be incorporated into outcomes research. Unfortunately, this can only help address sleep concerns after PICU discharge. To understand the role of sleep fragmentation during PICU admissions to PICS-p and outcomes would require a significant shift in bedside management, with a focus on monitoring sleep states using EEG or other wearable technology, and prioritizing sleep as an essential component of recovery and outcome.

## 6 Discussion

Despite the emerging evidence demonstrating various neurodevelopmental consequences of fragmented sleep in critically ill patients, optimal strategies for improving PICU sleep remain elusive. Recent advances in adult critical care have demonstrated that targeted interventions can significantly mitigate sleep disruption and its downstream effects (Dorsch et al., 2019; Zhang et al., 2024; Christina-Athanasia et al., 2024). Dynamic lighting systems that mimic natural circadian rhythms—providing cooler, brighter light (3000K/150 lux) during daytime hours and transitioning to warmer, dimmer tones (2,200 K/ < 10 lux) at night—have been shown to enhance melatonin secretion and enhance sleep quality (Marra et al., 2019; Hosseini et al., 2024). Complementary noise reduction strategies, particularly auditory masking using white or pink noise, effectively stabilize sound environments by reducing cortical activation during sleep, as evidenced by altered evoked potential amplitudes (Alegria et al., 2023). Similarly, cardiac ICU patients who used earplugs and eye masks overnight experienced improved sleep quality and a reduction in delirium incidence (Kiliç and Kav, 2023). Additionally, managing alarm fatigue is essential, as constant alarms can be detrimental to both patients and families. Positioning alarms away from patient rooms or utilizing ear protection devices, such as soft ear plugs, can significantly reduce these disturbances and improve sleep quality (Richardson et al., 2007).

The success of these environmental strategies has been amplified through bundled care frameworks. The Society of Critical Care Medicine's ABCDEF bundle, validated in over 15,000 adult ICU patients, reduced delirium incidence and mechanical ventilation duration through coordinated pain management, early mobility, and family engagement (Pun et al., 2019). Gorecki and Prasun further demonstrated that bundled sleep promotion protocols incorporating cycled lighting and noise reduction significantly decreased ICU length of stay and agitation scores (Gorecki and Prasun, 2024). Ongoing trials aim to build on this foundation by rigorously evaluating multi-component environmental interventions that target various aspects of the ICU environment (Alegria et al., 2023; Tronstad et al., 2024).

Moreover, technological advancements have paralleled these environmental interventions. Wearable EEG devices with machine learning capabilities now enable continuous sleep staging at the bedside, providing critical insights into NREM sleep preservation and delirium risk (van Twist et al., 2024). This approach provides real-time insights into sleep architecture, allowing for more precise differentiation between sleep and wake states and distinguishing between light and deep sleep. Such classification is particularly valuable for heavily sedated patients, allowing clinicians to tailor care schedules and minimize disruptions during restorative sleep phases (Markov et al., 2024). The ability to obtain continuous, high-resolution sleep data may enable clinicians to refine sedation protocols, optimize medication administration, and implement other interventions aimed at enhancing sleep quality (Ala-Kokko et al., 2022; Angelucci et al., 2025). The integration of such monitoring with bundled care protocols represents a promising frontier, as demonstrated by Knauert et al., who found preserved N2 sleep features were strongly associated with reduced mortality in delirious ICU patients (Knauert et al., 2018).

While adult ICU studies demonstrate the efficacy of sleep-promoting interventions, these strategies have not yet been rigorously evaluated in pediatric ICU settings. Given that up to 93% of pediatric ICU patients are reported by their parents to experience sleep disturbances—primarily attributed to nocturnal caregiving interactions and environmental disruptions (Burger et al., 2024)—the absence of tailored, evidence-based sleep interventions in this population represents critical oversight. Compared to adults, children require substantially more slow-wave sleep to support critical processes (Bertrand et al., 2020). As a result, their underdeveloped stress response systems heighten vulnerability to the adverse effects of sleep fragmentation (Kalvas et al., 2023). Communication limitations further complicate pain and sedation assessment in pediatric patients, elevating the risk of both under- and oversedation (Herrup et al., 2017; Kudchadkar et al., 2014). Current analgesia-first sedation approaches, though theoretically sound, suffer from inconsistent implementation due to limited pediatric pharmacodynamic data and staffing constraints (Shajan et al., 2023; Budic et al., 2023). Moreover, adult-derived strategies require significant modifications for pediatric use. Even promising adult-derived interventions face pediatric-specific barriers: circadian lighting must accommodate light sensitivity variations across ages, noise reduction strategies must balance alarm safety with sensory overload prevention, and bundled care requires resource-intensive implementation challenges (Richardson et al., 2007; Michel et al., 2022; Kawai et al., 2017).

Emerging pediatric-specific bundles demonstrate both the potential and limitations of current approaches. The modified ABCDEFGH bundle achieved a clinically meaningful 32% reduction in delirium incidence through its innovative two-phase system: utilizing noise/light shielding for sedated patients and parent-facilitated circadian entrainment for awake children (Engel et al., 2022). Similarly, the RESTORE Resilience (*R*^2^) bundle attained 63% protocol compliance by integrating cycled light/sound modulation with parental sleep diaries, showing significant improvements in daytime activity consolidation prior to extubation (Curley et al., 2024a). However, these small-scale feasibility studies remain without multicenter validation. Current research predominantly focuses on short-term outcomes, neglecting longitudinal neurodevelopmental consequences (Herrup et al., 2017). Pediatric trials face inherent challenges, including developmental variability in cognition, behavior, and organ maturation, which complicate study design, intervention feasibility, and outcome assessment (Herrup et al., 2017; Kudchadkar et al., 2014).

Designing effective interventions also requires consideration of pediatric chronotypes and developmental variation in sleep-wake cycling—from the polyphasic patterns of infants to the circadian shifts seen in adolescents. As a result, there remains a critical need to develop evidence-based approaches that not only promote sleep in pediatric ICU patients but also address the multifaceted consequences of sleep fragmentation while supporting neurodevelopmental recovery (Lee et al., 2023; Egbuta and Mason, 2021).

## 7 Future directions

To advance pediatric sleep science within critical care, the field must undergo a paradigm shift: sleep should be recognized not as a passive correlate of sedation but as a distinct, measurable physiologic state with vital implications for recovery and neurodevelopment. This reconceptualization necessitates the routine use of objective monitoring tools such as wearable EEG devices—compact, non-invasive headsets capable of continuously recording brain activity at the bedside— to distinguish between pharmacologically induced sedation and restorative sleep. To operationalize this shift, multicenter pilot trials must be designed to evaluate pediatric-adapted bundled interventions (Curley et al., 2024a). These studies should adopt objective sleep metrics—such as EEG-confirmed N3/SWS duration—as primary outcomes while incorporating standardized delirium assessments and tailoring interventions for family-centered care delivery. While modest in scope, such trials represent a foundational effort to elevate sleep to a neuroprotective priority in pediatric critical care. Moreover, longitudinal research should bridge the current gap between pediatric ICU sleep disruption and long-term outcomes by expanding the PICS-p cohort study to correlate in-hospital sleep architecture with neurodevelopmental trajectories post-discharge, while integrating sleep hygiene interventions into post-PICU follow-up clinics through multidisciplinary models (Ducharme-Crevier et al., 2021). Given the heightened neurodevelopmental vulnerability of critically ill children, incorporating assessments of sleep disruption into post-discharge follow-up protocols may help identify those at greatest risk for long-term cognitive and behavioral impairments. Furthermore, implementation science approaches are essential for translating research into practice. This includes developing consensus-based pediatric sleep promotion protocols, integrating sleep monitoring dashboards into electronic health records, and deploying targeted staff training in circadian rhythm entrainment and sleep preservation strategies. Dedicated funding mechanisms and multicenter collaborative networks will be vital to ensuring these interventions are scalable and adaptable across diverse PICU environments. In doing so, we shift from merely documenting sleep disruption to actively safeguarding the restorative processes essential to children's long-term health and developmental trajectories.

## 8 Conclusion

Sleep in the pediatric intensive care unit is not merely a passive state of recovery but rather an essential and active biological process integral to healing, neurodevelopment, and long-term recovery. Sleep fragmentation remains an underrecognized contributor to adverse outcomes such as delirium and Pediatric Post-Intensive Care Syndrome, yet tailored strategies to protect sleep in critically ill children are still lacking. While adult ICU models provide a foundation, pediatric-focused interventions must address the unique neurodevelopmental vulnerabilities of this population. Developing tailored PICU protocols that prioritize sleep promotion, minimize environmental disruptions, and actively engage families could profoundly improve children's neurodevelopmental trajectories and quality of life. Advancing this field can begin with pediatric-specific pilot trials to generate objective evidence, refine interventions, and establish sleep as a measurable, modifiable vital sign in critical care. By fostering robust research, clinical innovation, and multidisciplinary collaboration, the pediatric critical care community can transform sleep from an overlooked aspect of care into a central pillar of neuroprotective recovery.
